# The LaserSAFE technique for margin assessment during radical prostatectomy: a feasibility study

**DOI:** 10.1111/bju.70092

**Published:** 2025-11-27

**Authors:** Ricardo Almeida‐Magana, Larissa Sena Teixeira Mendes, Eoin Dinneen, Tarek Al‐Hammouri, Aiman Haider, Anna Silvanto, Alex Freeman, Nicholas Roberts, Louise Dickinson, Chun Wah So, Zafer Tandogdu, Benjamin W. Lamb, Nikhil Mayor, Mathias Winkler, Hashim Ahmed, Greg Shaw

**Affiliations:** ^1^ Division of Medicine and Interventional Science, Department of Targeted Intervention, Faculty of Medical Sciences University College London London UK; ^2^ Division of Medicine, Centre for Medical Imaging University College London London UK; ^3^ Department of Urology University College London NHS Foundation Trust London UK; ^4^ Department of Pathology University College London NHS Foundation Trust London UK; ^5^ Department of Radiology University College London NHS Foundation Trust London UK; ^6^ Department of Urology Barts Health NHS Trust London UK; ^7^ Barts Cancer Institute Queen Mary University of London London UK; ^8^ Department of Surgery and Cancer, Faculty of Medicine Imperial College London London UK; ^9^ Department of Urology Imperial College Healthcare National Health Service (NHS) Trust London UK; ^10^ Division of Cancer Surgery Peter MacCallum Cancer Centre Melbourne Victoria Australia

**Keywords:** feasibility study, fluorescence confocal microscopy, intraoperative margin assessment, prostate cancer, radical prostatectomy

## Abstract

**Objectives:**

To assess the feasibility of conducting a multicentre trial comparing NeuroSAFE with a novel technique based on confocal laser microscopy (LaserSAFE) and evaluate the diagnostic performance of LaserSAFE for real‐time surgical margin assessment.

**Patients and Methods:**

This was a non‐randomised, prospective feasibility study conducted at a high‐volume academic UK centre (ClinicalTrials.gov identifier: NCT06398470). Patients with localised prostate cancer (clinical T2–T3a N0 M0) scheduled for robot‐assisted radical prostatectomy and deemed unsuitable for bilateral intrafascial nerve sparing (NS) based on a multidisciplinary plan were included. LaserSAFE imaging was performed in the operating room after which the NeuroSAFE technique results guided NS decisions. Pathologists, blinded to NeuroSAFE and final histology, retrospectively evaluated LaserSAFE images. Diagnostic accuracy metrics and concordance between modalities were calculated. Feasibility was assessed based on recruitment rate and the ability to activate additional sites.

**Results:**

A total of 20 patients were recruited at a single site within 12 months of recruitment start. However, expansion to additional centres was not feasible due to limitations in implementing the NeuroSAFE protocol. LaserSAFE achieved a sensitivity of 0.91 (95% confidence interval [CI] 0.59–1.00) and specificity of 1.00 (95% CI 0.88–1.00) for detecting positive surgical margins ≥0.5 mm. Cohen's kappa demonstrated strong agreement with NeuroSAFE and final pathology. LaserSAFE was completed within a median of 7 min, significantly shorter than the 63 min required for NeuroSAFE. Limitations include the small sample size, single‐centre setting, and lack of intraoperative decision‐making based on LaserSAFE findings.

**Conclusion:**

While a multicentre study based on NeuroSAFE as a comparison was not achievable, LaserSAFE proved to be a rapid and accurate alternative for intraoperative margin assessment. These findings support the design of a larger trial in which NS decisions are informed by LaserSAFE, with a view to broadening access to real‐time margin assessment.

**Abbreviations**
CLM: confocal laser microscopy
CPG: Cambridge Prognostic Group
EPE: extraprostatic extension
IQR: interquartile range
mpMRI: multiparametric MRI
NS: nerve sparing
NVB: neurovascular bundle
PCa: prostate cancer
(N)(P) PV: (negative) (positive) predictive value
PSM: positive surgical margin
(RA)RP: (robot‐assisted) radical prostatectomy

## Introduction

Functional side effects after robot‐assisted radical prostatectomy (RARP) are mainly the result of damage to surrounding tissues rather than the removal of the organ per se [1, 2]. Given the protracted natural history of localised prostate cancer (PCa), many patients survive for more than two decades after treatment [3]. Therefore, developing strategies to minimise unwanted side effects is imperative and is one of the priorities outlined in the recent Lancet Commission on PCa [4].

The most impactful decision during RARP is whether to perform a nerve‐sparing (NS) technique or not, as this significantly influences functional outcomes, particularly erectile function and urinary continence [5]. NS can be graded according to the degree of preservation of the periprostatic fascia, commonly described as intrafascial, interfascial, or non‐NS (extrafascial) techniques. Intrafascial dissection involves maximal preservation of the neurovascular bundles (NVBs) and is associated with the best functional outcomes, whereas interfascial and extrafascial involve progressively wider excisions with a corresponding reduction in postoperative erectile function recovery [1, 5].

Traditionally, clinical assessment, DRE, and biopsy results were used to inform the decision on which NS approach is best for a particular patient, but in most settings this decision is taken without a systematic approach [6]. Multiparametric MRI (mpMRI) has transformed PCa diagnostic pathways and is increasingly used to inform NS surgical planning, but diagnostic performance varies and can suffer from both poor sensitivity and specificity, as is highlighted by a recent systematic review [7]. This means that in equivocal cases, patients are often excluded from NS procedures [8].

Intraoperative frozen‐section analysis of the NVB adjacent to the prostate margin (NeuroSAFE) can accurately detect positive surgical margins (PSMs) [9] and our team recently reported the first randomised controlled trial evaluating this technique, which demonstrated clinically significant benefits in 12‐month erectile function and 3‐month urinary continence outcomes following RARP [10]. Despite these benefits, NeuroSAFE is an expensive and time‐consuming addition to RARP. The technical requirements, logistical processes, and histological expertise may not be available in all units thus making implementation of the NeuroSAFE technique challenging [11]. Given the constraints on healthcare resources and the anticipated rise in PCa incidence, alternative approaches to NeuroSAFE may be necessary to broaden access to the benefits of intraoperative NS decision‐making [12].

We previously described the LaserSAFE technique for intraoperative *en face* surgical margin assessment of intact RP specimen using confocal laser microscopy (CLM; Histolog® Scanner, SamanTree Medical SA, Lausanne, Switzerland) [13]. This method is faster than NeuroSAFE and can be performed in the operating theatre, minimising procedural delays. Preliminary studies by our group and others indicate that histopathological interpretation of CLM images achieve high diagnostic accuracy compared to final paraffin‐embedded histopathology [14]. However, these studies have been small, or retrospective, and not compared to the current intraoperative ‘gold standard’ of frozen section [15].

Here, we present the results of a prospective, controlled, feasibility study to evaluate the accuracy of the LaserSAFE technique against NeuroSAFE.

## Patients and Methods

### Study Design

This was a non‐randomised prospective feasibility study designed to be conducted across at least two academic urology centres in the UK. Eligible participants were patients with localised PCa (clinical T2–T3a N0 M0) scheduled for RARP and deemed unsuitable for intrafascial NS on at least one side, based on preoperative mpMRI and multidisciplinary team recommendations.

Eligibility was determined during a preoperative planning meeting involving a team of radiologists with expertise in prostate mpMRI and a RARP surgeon, who assessed mpMRI images alongside clinical and biopsy data to recommend the appropriate grade of NS considered safe as we previously described [16]. Full eligibility criteria are detailed in the study protocol (Supporting Information). Exclusion criteria included radiographic evidence of rectal or seminal vesicle invasion, or previous PCa treatment (focal, androgen‐deprivation therapy, external‐beam radiotherapy).

Eligible patients were approached by clinical staff and offered a patient information sheet during their pre‐surgical consultation. Participants signed an informed consent form before enrolment. The protocol was developed with input from patient and public involvement events conducted during the NeuroSAFE PROOF trial (ClinicalTrials.gov identifier: NCT03317990) [10], specifically eligibility criteria was expanded to include all patients independent of their preoperative erectile function based on their recommendations. The study was registered on ClinicalTrials.gov (NCT06398470), sponsored by University College London, and approved by the London Surrey – Borders Research Ethics Committee (24/LO/0061). This was an investigator led study, but SamanTree Medical Inc. provided funding for consumables and lease of the Histolog Scanner but was not involved in the development, conduct, or analysis of the trial.

### Procedures

The RARP was performed using the da Vinci® Robot System (Intuitive Surgical Inc., Sunnyvale, CA, USA) by surgeons with experience in ≥100 prior cases. Access and surgical technique (anterior/preperitoneal space sparing) were performed according to surgeon preference. All patients received bilateral NS starting in the intrafascial plane independent of the mpMRI informed preoperative plan. Where technical challenges precluded an intrafascial dissection, interfascial or extrafascial NS was performed and the reason documented.

Immediately after the prostate was removed, the specimen was scanned in the operating room using the LaserSAFE technique (CLM with the Histolog scanner) as follows: the prostate was stained with Histolog Dip®, and three images (one posterior and one from each posterolateral aspect, left and right) were acquired in the operating theatre. De‐identified images were stored on the Histolog scanner for subsequent review and the specimen was transported to the pathology laboratory where the NeuroSAFE technique was performed. LaserSAFE images did not inform surgery in any way, instead the NeuroSAFE technique was used to inform NS as previously described by our group and others (see Appendix S1) [16, 17]. To accurately quantify the time associated with each technique, a dedicated case report form was completed prospectively for all participants. The precise start and end times of each procedural step were recorded by the operating team, allowing calculation of the total duration of both the LaserSAFE and NeuroSAFE components (see Appendix S1 for the reporting pro forma).

The decision for secondary resection of the full ipsilateral NVB was taken if NeuroSAFE identified Gleason Grade 3 adenocarcinoma exceeding 2 mm in linear length, any Gleason Grade 4 or 5 adenocarcinoma, or multifocal involvement (tumour present in more than one section from the same side). Resection involved *en bloc* removal of residual soft tissue adjacent to the affected NVB, bounded by Denonvilliers’ fascia medially, pararectal fat laterally, the vascular pedicle cranially, and the urethrovesical anastomosis caudally. Final histopathology was assessed using paraffin‐embedded analysis.

**Fig. 1:**
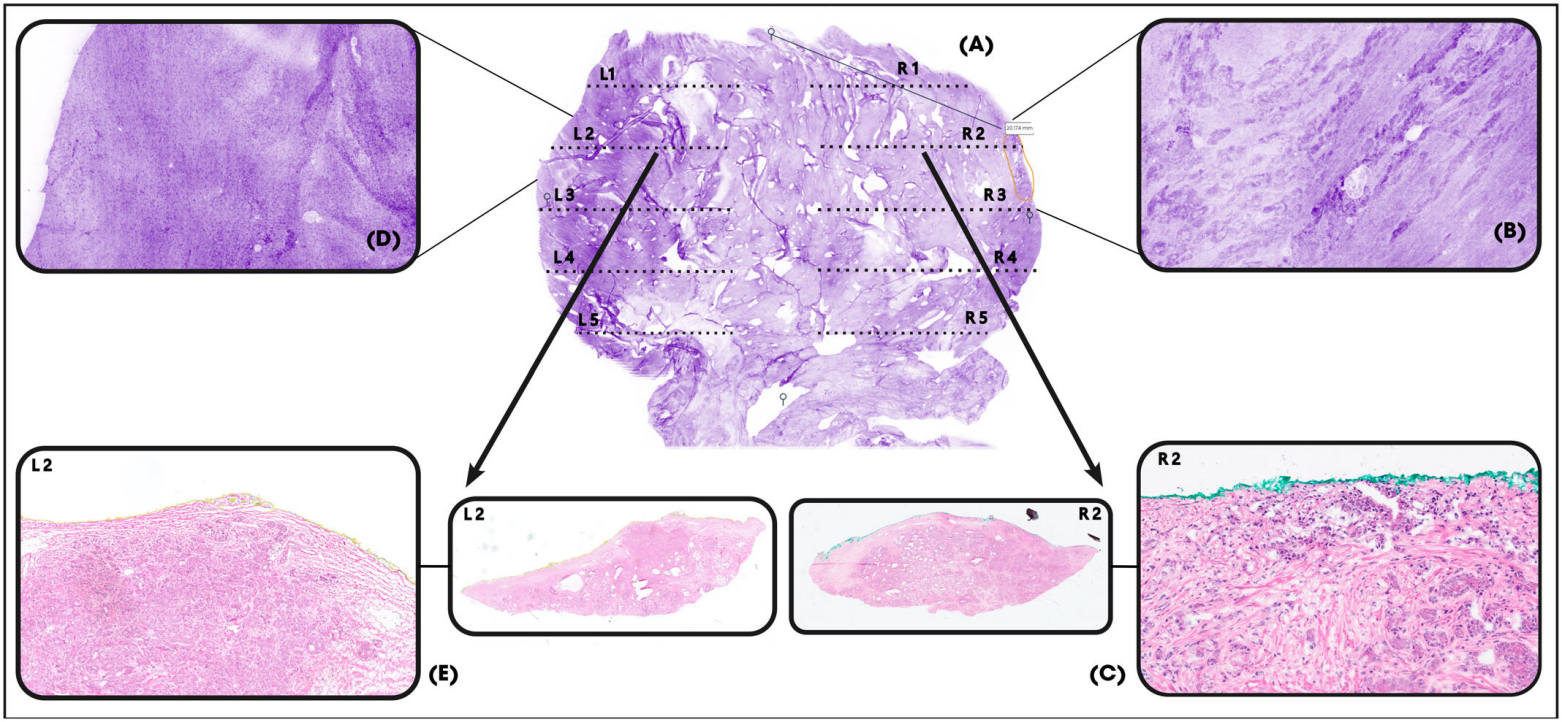
Comparison of *ex vivo* CLM and FS analysis for evaluating surgical margins. (A) CLM *en face* scan of the posterior prostatic surface, indicating the locations of the sampled areas during FS. (B, C) PSM: (B) An CLM image showing prostate cancer glands, and (C) the corresponding FS H&E stain confirming a PSM. (D, E) Negative margin: (D) CLM image with smooth fibromuscular stroma, and (E) the corresponding FS H&E stain confirming a negative margin. FS, frozen section; H&E, haematoxylin and eosin stain.

LaserSAFE images were securely stored in the Histolog Scanner system and retrospectively reviewed by consultant uropathologists with expertise in prostate confocal microscopy interpretation. Following completion of all paraffin‐embedded histological analyses, and after a 2‐month wash‐out period, a structured review process was implemented where the evaluating pathologists examined the LaserSAFE images in randomly ordered batches, with all patient identifiers removed. Pathologists evaluating LaserSAFE images were blinded to results of NeuroSAFE and final histopathology. Figure 1 shows a comparison between NeuroSAFE and LaserSAFE assessments.

Clinical follow‐up adhered to each centre's standard protocols and complications were monitored (adverse events) for 30 days after surgery.

### Objectives

The primary objective was to assess the feasibility of recruiting patients into a study comparing LaserSAFE and NeuroSAFE techniques. The primary endpoint was the proportion of eligible patients successfully recruited within the recruitment period, including at least one participant from a second NHS site.

Secondary objectives included evaluating concordance between LaserSAFE and NeuroSAFE regarding secondary resection recommendations, diagnostic accuracy of LaserSAFE (sensitivity, specificity, positive predictive value [PPV], negative predictive value [NPV]) for PSM detection, prevalence of PSMs in the cohort, rates of PSMs outside the posterolateral analysis field, and detailed prospectively collected timing parameters for NeuroSAFE and LaserSAFE techniques. Exploratory endpoints included mpMRI recommendation accuracy and margin detection accuracy for margins ≥0.5 mm.

### Statistical Analysis

A convenience sample of 20 patients was selected for this feasibility study, to demonstrate potential recruitment to a full study and provide information as outlined in the secondary objectives for study design including technical elements of CLM. For secondary outcomes, diagnostic accuracy metrics (sensitivity, specificity, PPV, NPV) and 95% CIs were calculated. Results agreement was assessed using Cohen's kappa (κ) with 95% CIs. Descriptive statistics were applied to remaining data. Analyses were based on complete case analysis (patients who had surgery) and performed in R (version 4.4.1; R Foundation for Statistical Computing, Vienna, Austria) using ‘epiR’ and ‘irrCAC’ packages.

## Results

### Recruitment and Cohort Characteristics

Between May and December 2024, 21 participants out of 195 eligible patients were recruited from a single centre (University College London Hospital). Two additional UK NHS centres were invited to join the study but could not be activated because the NeuroSAFE technique could not be performed by their histopathology departments. Main concerns were lack of skilled technicians trained in the use of cryotome and limited expert pathology workforce. In addition, the participating urology departments expressed concern that incorporating NeuroSAFE could cause intraoperative delays, potentially preventing completion of three RARP procedures within a single surgical list.

**Fig. 2:**
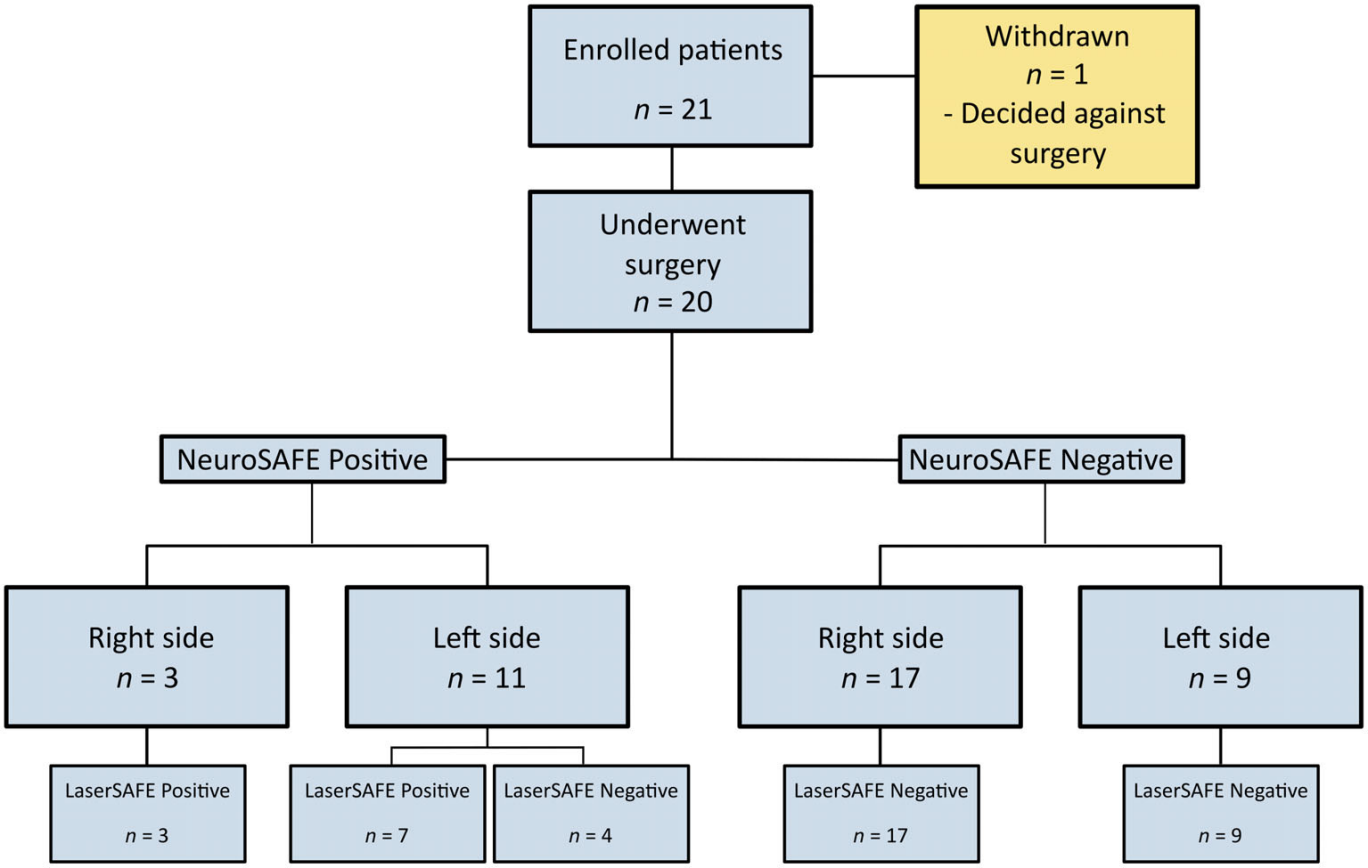
Participant flow diagram of the LaserSAFE feasibility study.

One recruited patient decided against surgery after enrolment and was withdrawn see study flow diagram (Fig. 2). Baseline, radiological, and surgical characteristics of the cohort are presented in Table 1. Most preoperative mpMRI scans were of acceptable quality for interpretation, but 35% of scans were deemed of low quality. Seven patients had a score ≥3 on the MRI‐based Likert scale for extraprostatic extension (EPE) on the right side and 12 on the left side. In all, 19 patients had Cambridge Prognostic Group (CPG) ≥2 PCa. One patient, initially classified as CPG1, opted for RARP due to high‐volume International Society of Urological Pathology (ISUP) Grade Group 1 disease and discordant mpMRI findings that suggested more aggressive disease. Final pathology confirmed upgrading in this case, with PSMs necessitating secondary resection on one side.

**Table 1:** Baseline and surgical characteristics of the 20 patients.

Characteristic	Value
Age, years, median (range)	59 (51–73)
Body mass index, kg/m^2^, median (range)	26.36 (21.55–32.24)
Ethnicity, *n* (%)	
White	15 (75)
Black	4 (20)
Asian	1 (5.0)
Prostate volume, mL, median (range)	32 (18–113)
MRI grade (PIQUAL‐2), *n* (%)	
1	7 (35)
2	9 (45)
3	4 (20)
T stage (MRI based), *n* (%)	
T1	1 (5.0)
T2	9 (45)
T3	10 (50)
Biopsy Grade Group, *n* (%)	
Grade Group 1	1 (5.0)
Grade Group 2	11 (55)
Grade Group 3	8 (40)
CPG classification, *n* (%)	
1	1 (5.0)
2	6 (30)
3	3 (15)
4	10 (50)
Type of surgery, *n* (%)	
Anterior approach	18 (90)
Preperitoneal space sparing	2 (10)
Total surgical time, min, median (range)	175 (149–248)
LaserSAFE time, min, median (range)	7.00 (3.00–10.00)
NeuroSAFE time, min, median (range)	63 (43–92)
Deeper levels required during NeuroSAFE, *n* (%)	8 (40)
Number of deeper levels required, median (range)	1.50 (1.00–4.00)

PIQUAL‐2, Prostate Imaging Quality Scoring System, version 2.

Two RARPs were performed through a preperitoneal space‐sparing approach while the rest were performed using an anterior approach. The NeuroSAFE technique added a median (interquartile range [IQR]) of 63 (53.5–72.25) min to the duration of RARP. The median (IQR) time taken to perform the LaserSAFE technique was 7 (5.0–8.0) min.

### Surgical Margin and NS Outcomes

The MRI, NeuroSAFE and final NS results are detailed in Table 2 presented by prostate side. Overall, out of 40 sides of the prostate examined there were 14 (35%) PSMs and these were more frequent on the left side. All PSMs identified by NeuroSAFE were concordant with final paraffin‐embedded histopathology. In three cases, NeuroSAFE demonstrated a very short PSM involvement (<0.1 mm linear length of Gleason Grade 3 tumour). In these instances, the corresponding NVB was preserved, as the findings did not meet the secondary resection threshold. Notably, none of these three sub‐secondary‐resection threshold margins were detected by LaserSAFE.

**Table 2:** Side‐specific outcomes: MRI planning, NeuroSAFE, and LaserSAFE results.

Characteristic	Right side, *N* = 20	Left side, *N* = 20
Likert score for EPE, *n* (%)		
1	8 (40)	3 (15)
2	5 (25)	5 (25)
3	4 (20)	3 (15)
4	3 (15)	8 (40)
5		1 (5.0)
NS MRI‐based recommendation, *n* (%)		
Intrafascial	8 (40)	6 (30)
Interfascial	8 (40)	5 (25)
Wide resection	4 (20)	9 (45)
NeuroSAFE result, *n* (%)		
Negative	17 (85)	9 (45)
Positive	3 (15)	11 (55)
Secondary resection actioned according to NeuroSAFE result	2 (10)	7 (35)
Final NS status, *n* (%)		
Intrafascial NS	16 (80)	13 (65)
Interfascial NS	2 (10)	–
No NS	2 (10)	7 (35)
LaserSAFE result, *n* (%)		
Negative	17 (85)	13 (65)
Positive	3 (15)	7 (35)
Distance from apex to nearest gland, mm, median (range)	11.0 (6.0–25.0)	12.0 (1.0–21.0)
PSM size in the sagittal plane, mm, median (range)	5.00 (1.00–6.50)	7.00 (5.00–13.00)
PSM size in the coronal plane, mm, median (range)	6.5 (1.5–20.0)	7.00 (5.00–10.00)

In two cases, small PSMs on NeuroSAFE that did not warrant resection as per protocol, the LaserSAFE analysis identified a large PSM area. In these two cases final pathology confirmed a longer length of PSM than was initially identified during NeuroSAFE.

### Diagnostic Performance of LaserSAFE

Accuracy metrics revealed high concordance between LaserSAFE and the reference standards (NeuroSAFE and final pathology), as summarised in Table 3. For all PSMs (irrespective of length), LaserSAFE demonstrated a sensitivity of 0.71 (95% CI 0.42–0.92). When applying a ≥0.5 mm threshold, only one PSM was missed, sensitivity of 0.91 (95% CI 0.59–1). Inter‐modality agreement was robust, with Cohen's κ indicating strong concordance for margin detection (any size or ≥0.5 mm) and for secondary resection recommendations κ.

**Table 3:** Accuracy metrics and diagnostic value of LaserSAFE vs final histopathology.

	LaserSAFE all margins	LaserSAFE for margins >0.5 mm	Recommendation for secondary resection	MRI planning for all margins
	Value (95% CI)	Value (95% CI)	Value (95% CI)	Value (95% CI)
Sensitivity	0.71 (0.42–0.92)	0.91 (0.59–1)	0.89 (0.52–1)	0.64 (0.35–0.87)
Specificity	1.00 (0.87–1)	1.00 (0.88–1)	0.94 (0.79–0.99)	0.85 (0.65–0.96)
PPV	1.00 (0.69–1)	1.00 (0.69–1)	0.80 (0.44–0.97)	0.69 (0.39–0.91)
NPV	0.87 (0.69–0.96)	0.97 (0.83–1)	0.97 (0.83–1)	0.81 (0.62–0.94)
κ coefficient	0.76 (0.54–0.98)	0.94 (0.80–1)	0.79 (0.56–1)	0.50 (0.20–0.79)

### Final Pathology and Clinical Outcomes

Table S1 outlines final pathology results. Most cases (14/20) exhibited organ‐confined disease (pathological T2). Among the nine patients who underwent secondary resection of the NVB, eight achieved negative posterolateral margins, in the remaining case the PSM extended more medially than the area sampled by NeuroSAFE. In three of the nine NVBs resected PCa was identified.

Bilateral intrafascial NS was accomplished in eight patients (40%). Only one patient required bilateral non‐NS surgery. Three patients experienced adverse events within 90 days after surgery, all were classified as Grade 2 (anastomotic leak, UTI, anterior abdominal haematoma), but none were considered related to the intervention. There were no serious adverse effects or deaths.

## Discussion

To our knowledge, this is the first prospective study evaluating the accuracy of CLM on intact RP specimens (LaserSAFE) against the NeuroSAFE technique for intraoperative margin assessment. Although the study met its recruitment target at a single centre within the specified timeframe, it failed to demonstrate feasibility across multiple hospitals in the UK. At two high‐volume RARP referral centres that were invited, practical and technical obstacles to the NeuroSAFE technique prevented the activation of the LaserSAFE feasibility study protocol. Nevertheless, recruitment at the lead site was rapid, and LaserSAFE procedures were implemented with minimal logistical challenges. We demonstrated a high concordance between LaserSAFE and NeuroSAFE results, and almost complete concordance between the LaserSAFE assessment recommendations and the current intraoperative reference standard, the NeuroSAFE technique.

The NeuroSAFE technique, developed at the Martini‐Klinik (Hamburg, Germany) over two decades ago [18], has been shown to be accurate in guiding NS decisions without compromising oncological safety [9, 19]. The NeuroSAFE PROOF randomised trial recently confirmed its functional benefits in a multicentre setting [10]. Despite this, widespread adoption remains limited due to the procedural demands of NeuroSAFE, including the need for dedicated pathology infrastructure and specialised personnel [11, 20]. Centres capable of performing NeuroSAFE typically have on‐site histopathology facilities and a high volume of frozen section requests, which help streamline workflow and reduce delays. In the current context of constrained healthcare resources and increasing operational costs, replicating this is challenging even for tertiary referral centres. Our study supports previous observations that establishing NeuroSAFE outside of highly specialised centres is challenging, underscoring the need for more practical alternatives such as LaserSAFE and other new technologies [21].

A previous study used the Histolog Scanner to assess RP specimens and compared the results to NeuroSAFE, with similar accuracy results. However, their technique required the posterolateral areas of the specimen to be cut up before analysis [22]. This introduces the potential for capsular retraction, which raises the possibility of false‐positive PSMs and makes it difficult to orient the prostate tissue slices. This approach also requires the direct involvement of a histopathologist in specimen processing. In contrast, the LaserSAFE technique allows for intact specimen imaging and can be conducted by trained surgical staff with minimal disruption to the operating room workflow. This represents a significant procedural advantage and may enhance accessibility to real‐time margin assessment across diverse clinical settings.

A parallel study at another UK centre evaluated the accuracy of CLM by imaging the entire prostatic surface, taking six *en face* images using the Histolog Scanner [23, 24]. Although the preliminary report showed overall sensitivity for PSMs was lower than in our study, sensitivity increased with a PSM length of 3 mm (71%). Several key differences may explain these findings. First, the posterolateral margins assessed in our study were typically smooth and free of overlying tissue following intrafascial dissection. This facilitates image interpretation as the images are typically devoid of features and mostly show smooth connective tissue in cases of negative margins (Fig. 1). Second, electrocautery artefacts at the apex, base, and anterior surfaces complicate CLM interpretation because image quality is decreased by necrotic tissue.

A novel finding of our study is the average length of PSM of 7 mm observed in CLM *en face* images. This represents, to our knowledge, the first reported measurement of this characteristic in *en face* margins. While the clinical significance of this specific *en face* PSM length is yet to be determined, a threshold of 3 mm for PSM length in final histopathology has been established as a predictor of worse oncological outcomes [25]. This finding may therefore provide an initial foundation for investigating whether a similar length threshold exists for PSMs identified via *en face* imaging and whether it correlates with patient prognosis.

Although mpMRI‐based recommendations had lower accuracy for predicting PSMs than both real‐time margin analysis techniques, there was still a good correlation between EPE Likert scores and PSMs (Fig. S1). Though most mpMRI scans were considered of good quality for interpretation, 35% were deemed of low quality, which could have impacted the accuracy of the findings. Furthermore, while MRI can grade the likelihood of EPE, which is not perfectly related to a PSM, the strong evidence that an EPE Likert score of 5 was always associated with a positive NeuroSAFE result suggests we may consider excluding these prostate lobes from future studies using intraoperative margin assessment.

Although bilateral NS rates were lower than those reported in the NeuroSAFE PROOF trial (40% vs 82%) [10], this reflects our deliberate inclusion of patients for whom bilateral intrafascial NS would not otherwise have been considered. Despite this, we were still able to change six prostate sides from non‐NS to intrafascial and 11 from interfascial to intrafascial. This supports the notion that real‐time margin assessment may offer more value in cases where NS is otherwise contraindicated [15]. That said, PSMs can occur in any patient, and expanding access to intraoperative assessment with a more convenient alternative could benefit a broader population.

Other novel technologies for real‐time assessment of surgical margins, such as Raman spectroscopy [26] and the Vivascope 2500M‐G4 device [27], also achieve good accuracy in detecting PSMs and offer faster workflows than NeuroSAFE. However, both have smaller fields of image acquisition, requiring tissue processing or the use of smaller biopsies. A key advantage of the LaserSAFE technique is that scanning intact specimens simplifies the procedure so that any member of the team can obtain high‐quality images with minimal training. A recent study showed that intraoperative margins can be assessed *in* vivo and *ex vivo* in the peri‐prostatic tissue using prostate‐specific membrane antigen‐labelled near‐infrared fluorescence, which offers a possible alternative or could be complementary to our technique [28].

The learning curve for pathologists interpreting CLM is unknown. We acknowledge the pathologist participating in this study had extensive experience with the imaging modality, and this may not be the case at other centres. However, training resources are available on‐line [29], and this will increase as interest in this technology grows. A formal evaluation of this critical aspect is warranted to ensure patient safety when making decisions based on CLM imaging. Although this study did not formally quantify interpretation time, our experience suggests that image review generally takes <10 min, and even less with increasing reader experience and high‐quality imaging. This is comparable to the time required to assess NeuroSAFE slides but avoids the 30–45 min of additional specimen processing associated with frozen‐section preparation. Furthermore, LaserSAFE does not require additional paraffin blocks, thereby reducing both consumable costs and workload for histopathology departments.

Several limitations are to be acknowledged. This was a feasibility study with a small sample size designed to evaluate recruitment and logistical challenges. As such, the accuracy metrics should be interpreted with caution due to the wide CIs. Second, although NeuroSAFE was challenging to implement in the centres we approached, other institutions with different pathology infrastructure may find it more feasible to adopt. Finally, intraoperative decision‐making was based exclusively on NeuroSAFE, so the oncological safety and clinical impact of decisions guided by LaserSAFE remain untested. A future trial should evaluate the outcomes of real‐time decision‐making based directly on LaserSAFE findings; the accuracy metrics from this trial will help inform sample size calculations for this subsequent study.

We conclude that implementing a multicentre trial comparing LaserSAFE and NeuroSAFE was not feasible due to significant logistical and resource‐related challenges associated with the NeuroSAFE technique. Despite this, the LaserSAFE technique was easily integrated into clinical workflows at a high‐volume centre, with encouraging diagnostic performance and minimal procedural burden. These findings justify further investigation into LaserSAFE in a larger, prospective study where intraoperative decisions are based on LaserSAFE findings. This will help establish its clinical utility, safety, and impact on oncological and functional outcomes.

## Disclosure of Interests

Ricardo Almeida‐Magana and Greg Shaw have received support from SamanTree Medical SA to attend conferences; subsequent to the acceptance of this article Prof Shaw has taken up a paid medical consultancy for SamanTree and is now a shareholder. [Correction added after first online publication on 21 May 2026: The preceding sentence has been revised in this version.] Ricardo Almeida‐Magana receives salary from the following grant: Prostate Cancer UK MA‐CT20‐11. All other authors declare no conflicts of interest in relation to this manuscript.

## Funding

Samantree Medical SA provided funding for consumables and lease of the Histolog Scanner but had no role in the development, conduct, or analysis of the trial. The study conduct was supported by the Royal CSS‐CONS‐2021\11 grant. The methodology and protocol development were supported by the following grants: UK National Institute for Health Research (NIHR) Research for Patient Benefit PB‐PG‐1216‐20013, The Rosetrees Trust 566412, and the WEISS Health Challenge 2020.

## Supporting information


**Table S1.** Table of additional pathological characteristics and adverse events.
**Fig. S1.** The NeuroSAFE results by MRI EPE Likert score.
**Fig. S2.** The NeuroSAFE and LaserSAFE case report form.
