# Supplementary material for: The LaserSAFE technique for margin assessment during radical prostatectomy: a feasibility study
Source: BJU Int. 2025 Nov 27;138(Suppl 1):S22–30. doi: 10.1111/bju.70092 (PMC13314559; doi:10.1111/bju.70092)
Supplement: Supplementary file 1 — Table S1. Table of additional pathological characteristics and adverse events. Fig. S1. The NeuroSAFE results by MRI EPE Likert score. Fig. S2. The NeuroSAFE and LaserSAFE case report form. [file BJU-138-S22-s001.docx]

# Supplementary materials

Supplementary table 1:

Title: Table of additional pathological characteristics and adverse events

| **Characteristic** | **N = 20***^1^* |
| --- | --- |
| Prostate weight (grams) | 36 (26, 112) |
| Grade group on final paraffin analysis |  |
| Grade group 2 | 17 (85%) |
| Grade group 3 | 2 (10%) |
| Grade group 5 | 1 (5.0%) |
| Pathological T stage |  |
| pT2 | 16 (80%) |
| pT3a | 2 (10%) |
| pT3b | 2 (10%) |
| Positive margin converted to negative on the right (out of 3) | 1 (33%) |
| Positive margin converted to negative on the left (out of 11) | 7 (35%) |
| Cancer present in secondary resection (out of 9) | 3 (63%) |
| Adverse events (All were grade 2) | 3 (15%) |
| *^1^*Median (Min, Max); n (%) | |

Supplementary figure 1

Title: NeuroSAFE results by MRI EPE Likert Score


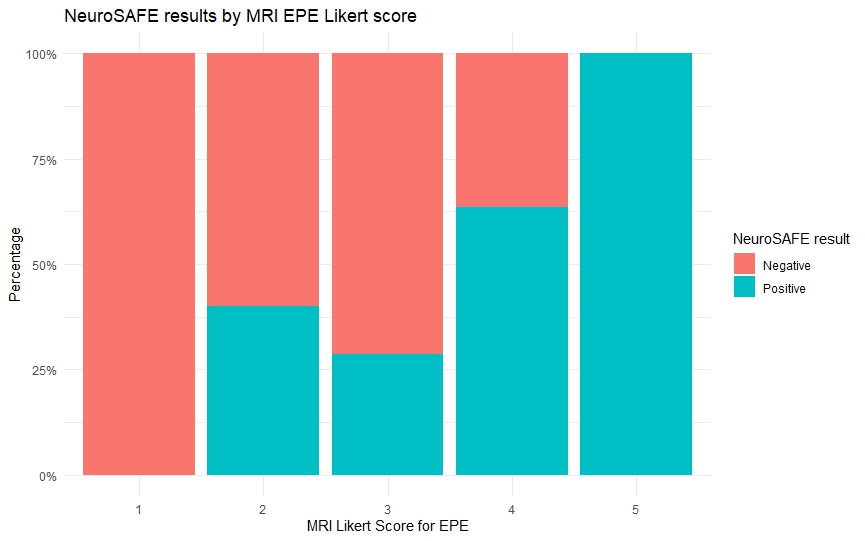


Caption: Chart showing the proportion of positive/negative NeuroSAFE results according to the MRI Likert Score for EPE

Supplementary Figure 2

Title: NeuroSAFE and LaserSAFE case report form

***Surgery capture sheet***

***Surgeon ____________________________***

***Date of Surgery ________________ Type of surgery ________________***

***DRE findings:***

| ***Right*** |  | ***Left*** |  |
| --- | --- | --- | --- |
| ***Not palpable*** |  | ***Not palpable*** |  |
| ***T2*** |  | ***T2*** |  |
| ***T3a*** |  | ***T3a*** |  |
| ***T3b*** |  | ***T3b*** |  |
| ***T4*** |  | ***T4*** |  |

***Timings:***

| ***Knife to skin*** |  | ***Docking finished*** |  |
| --- | --- | --- | --- |
| ***Specimen removed*** |  | ***LaserSAFE starts on 1^st^ side*** |  |
| ***LaserSAFE starts on 2^nd^ side*** |  | ***LaserSAFE ends*** |  |
| ***Specimen sent to pathology*** |  | ***Specimen arrives at pathology*** |  |
| ***Decision phoned to surgeon*** |  | ***Console finished*** |  |
| ***Skin closed*** |  |  |  |

***Quality of nerve spare***

| ***Right*** |  | ***Left*** |  |
| --- | --- | --- | --- |
| ***No nerve spare*** |  | ***No nerve spare*** |  |
| ***Interfascial nerve spare*** |  | ***Interfascial nerve spare*** |  |
| ***Intrafascial nerve spare*** |  | ***Intrafascial nerve spare*** |  |
|  |  |  |  |
| ***High release*** |  | ***High release*** |  |
| ***Low release*** |  | ***Low release*** |  |
|  |  |  |  |
| ***Secondary resection*** |  | ***Secondary resection*** |  |

***NeuroSAFE INFO CAPTURE SHEET***

Date and time specimen received at the laboratory _______________

Weight ____g Right side inked: **Green** Left side inked: **Orange** Defects: **Yellow**

**Right side** R1 Apex **Left side** L1 Apex

R2 ↓ L2 ↓

R3 ↓ L3 ↓

R4 ↓ L4 ↓

R5 ↓ L5 ↓

*R6 ↓ *L6 ↓

*R7 ↓ *L7 ↓

Base Base

*only when necessary because of large prostate, to ensure a maximum 5mm distance between sections.

**If positive:**

- **≤2mm G3 on a single section: No action required.**
- **>2mm G3, *any* G4, or cancer on multiple (>1) sections: Secondary resection/removal of the entire bundle.**

**FROZEN SECTION EXAMINATION**

| **Right side** | | **Left side** | |
| --- | --- | --- | --- |
| **R1** | **Positive for … .mm Gleason ( )** | **L1** | **Positive for ….mm Gleason (…. )** |
|  | **Narrowly Clear (<0.5mm)** |  | **Narrowly Clear (<0.5mm)** |
|  | **Clear** |  | **Clear** |
| **R2** | **Positive for … .mm Gleason ( )** | **L2** | **Positive for ….mm Gleason (…. )** |
|  | **Narrowly Clear (<0.5mm)** |  | **Narrowly Clear (<0.5mm)** |
|  | **Clear** |  | **Clear** |
| **R3** | **Positive for … .mm Gleason ( )** | **L3** | **Positive for ….mm Gleason (…. )** |
|  | **Narrowly Clear (<0.5mm)** |  | **Narrowly Clear (<0.5mm)** |
|  | **Clear** |  | **Clear** |
| **R4** | **Positive for … .mm Gleason ( )** | **L4** | **Positive for ….mm Gleason (…. )** |
|  | **Narrowly Clear (<0.5mm)** |  | **Narrowly Clear (<0.5mm)** |
|  | **Clear** |  | **Clear** |
| **R5** | **Positive for … .mm Gleason ( )** | **L5** | **Positive for ….mm Gleason (…. )** |
|  | **Narrowly Clear (<0.5mm)** |  | **Narrowly Clear (<0.5mm)** |
|  | **Clear** |  | **Clear** |
| **R6** | **Positive for … .mm Gleason ( )** | **L6** | **Positive for ….mm Gleason (…. )** |
|  | **Narrowly Clear (<0.5mm)** |  | **Narrowly Clear (<0.5mm)** |
|  | **Clear** |  | **Clear** |
| **R7** | **Positive for … .mm Gleason ( )** | **L7** | **Positive for ….mm Gleason (…. )** |
|  | **Narrowly Clear (<0.5mm)** |  | **Narrowly Clear (<0.5mm)** |
|  | **Clear** |  | **Clear** |
| **No resection ( )**  **Resect ipsilateral bundle ( )** | | **No resection ( )**  **Resect ipsilateral bundle ( )** | |

| **Deeper levels requested** | **Yes ( ) No ( )** |
| --- | --- |
| **Number of deeper levels** | **_________** |

- **Reported by: Dr**
- **Report was given to:**
- **Date of report:**
- **Time of report**
  - **Right side Left side**
